# Clindamycin-Based 3D-Printed and Electrospun Coatings for Treatment of Implant-Related Infections

**DOI:** 10.3390/ma14061464

**Published:** 2021-03-17

**Authors:** Tina Maver, Tinkara Mastnak, Mihela Mihelič, Uroš Maver, Matjaž Finšgar

**Affiliations:** 1 Institute of Biomedical Sciences, Faculty of Medicine, University of Maribor, Taborska ulica 8, SI 2000 Maribor, Slovenia; uros.maver@um.si; 2 Department of Pharmacology, Faculty of Medicine, University of Maribor, Taborska ulica 8, SI 2000 Maribor, Slovenia; 3 Faculty of Chemistry and Chemical Engineering, University of Maribor, Smetanova ulica 17, SI 2000 Maribor, Slovenia; tinkara.mastnak@um.si (T.M.); mihela.mihelic@student.um.si (M.M.)

**Keywords:** clindamycin, bioactive coating, 3D printed coating, electrospun coating, TiAlV, AISI 316LVM

## Abstract

This study presents the development and characterisation of two novel bioactive coatings deposited on TiAlV and AISI 316LVM substrates. The coatings were prepared using 3D printing and electrospinning. The 3D-printed coating consisted of the cellulose nanofibril suspension, alginate, and carboxymethylcellulose (CMC), while CMC and polyethylene oxide were used to prepare the electrospun coating. Both coatings were loaded with the antibiotic clindamycin (CLIN), which is a bacteriostatic lincosamide known for its activity against streptococci, staphylococci, pneumococci, Bacteroides species, and other anaerobes. Initial characterisation of the coatings was performed by attenuated total reflectance Fourier transform infrared spectroscopy, field emission scanning electron microscopy, and atomic force microscopy. Furthermore, the contact angle measurements, swelling rate, and biodegradability of the coatings were investigated. The released concentration of CLIN in PBS (pH = 7.4 at 25 °C) was determined by UV-VIS spectrophotometry. The coatings’ biocompatibility was determined using an MTT (3(4,5 dimethylthiazolyl-2)-2,5-diphenyltetrazolium bromide) assay using an osteoblast cell culture (hFOB 1.19, ATCC CRL 11372).

## 1. Introduction

The unprecedented aging of the world’s population and the growing prevalence of age-related conditions, such as osteoporosis and bone fractures, boosted the need for various medical implants that importantly influenced global healthcare costs. Since orthopaedic implants present the largest share of the international medical implant market [1], high-performance implantable biomaterials are in extremely high demand.

Orthopaedic implants for load-bearing and internal fixation are strongly bound to bones to ensure minimal movement between the host tissue and the implant. Furthermore, they possess load-bearing capabilities at the implantation site. Although the availability of such materials is high, only a few materials are biocompatible and thus capable of long-term success in implant applications. These materials include the titanium alloy Ti90Al6V4 (hereinafter TiAlV), surgical grade stainless steel (usually AISI type 316L), and cobalt-based alloys [2]. These materials exhibit excellent tensile strength, fracture toughness, and withstand fatigue stress with great success [3]. However, even an ideal artificial material can only be a compromise in response to mechanical strains, physicochemical degradation, aging, wear, and tear [4]. Moreover, all implanted medical devices suffer from recognised risks of device-related infections [5,6].

About 70% of implant-related infections are caused by Staphylococci species such as *Staphylococcus aureus* and *Staphylococcus epidermidis* [7]. These infections are difficult to treat because of the ability of the organisms to form biofilms [8]. Insufficient delivery of drugs to the infection site (possible in systemic drug administration) cannot reduce the formation of bacterial biofilms on the implants, making the treatment challenging [9]. Evidence suggests that coating of the implant surface effectively reduces the risk of infection after implantation. Therefore, methods are being developed to minimise the risk of implantable device infection by modifying the coating composition to decrease bacterial adhesion and minimise biofilm formation [10]. 

Three-dimensional (3D) printing enables the production of tailor-made scaffolds through computer-aided design (CAD) in a layer-by-layer manner [11,12]. An important advantage of fabricating implants using 3D printing compared to the traditional machining technology is the personalised real-time manufacturing of complex structures with high dimensional accuracy and short production cycles [13]. However, regardless of the basic manufacturing method, all implant coatings must be biocompatible and shall promote osteointegration after implantation. 

Hydrogels are hydrophilic polymeric networks capable of soaking up huge volumes of water while undergoing swelling and shrinkage, through which they facilitate controlled drug-release [14]. Hydrogels composed of natural polymers, such as gelatin, chitosan, alginate (ALG), and cellulose derivatives, have been extensively explored as scaffold materials due to their biodegradability, biocompatibility, and nontoxicity [15]. Moreover, hydrogels have been proven to enhance tissue regeneration and repair speed by mimicking the native extracellular matrix [13]. 

Nanofibers are fibrous structures with diameters less than 100 nm. These nanostructures possess a high surface area to volume ratio, high porosity, and small pore size, enabling favorable conditions for liquid adsorption in filter media, limiting bacterial penetration, and promoting wound healing [16]. Thus, it is not surprising that nanofibers have been studied as drug delivery systems in cancer treatment, surgical intervention, and tissue scaffolding [17]. Electrospinning is a unique technology characterised by operational simplicity and the ability to produce nanofibers on an industrial scale [18]. Furthermore, this technique allows the development of biocompatible nanofibers, which possess high loading efficiency and enable sustained and controlled drug release [16]. 

The coatings enable drug delivery directly to the infection site, which cannot be achieved by systemic administration due to off-target systemic unwanted side effects [19]. Despite the successful advances in preclinical studies, a remarkable discrepancy exists between the proposed approaches for antibacterial implant protection strategies (e.g., polymer-based medical implants, chemical additions to polymeric implant to control microbial growth) and their clinical applications (e.g., uncertainly of cost-effectiveness, infections are sometimes exacerbated by the implantation of polymeric medical implants, biofilm formation despite coatings) [20,21]. Only four technologies with reported clinical results are currently available in orthopaedics and traumatology [22]. Among other trends in the preparation of novel solutions in orthopedic implants also include additive manufacturing approaches of metal substrates [23,24]. Therefore, pursuing new fundamental research topics is crucial to successfully seal the gap between the newly invented materials and their clinical applications. 

The objective of this research was to develop novel antibiotic-functionalised coatings with a unique composition and study their applicability for the treatment of infections in hip implants. To achieve this, TiAlV and AISI 316LVM (medical grade stainless steel) metal substrates were used as models for hip prostheses and coated by two state-of-the-art techniques, namely electrospinning and 3D printing. Whereas electrospinning is known to produce material with a morphology often resembling 3D printing [25,26], with its reliance on computer-aided-design (CAD) modeling, it enables the preparation of coating with tailorable properties [27]. Many polymers made from biological sources offer natural hydrophilicity, biocompatibility [28], biodegradability, and regenerative characteristics (e.g., osteoinduction and osteointegration) [29], which makes them suitable for drug delivery applications. For this reason, sodium alginate, a water-soluble, non-toxic, and biocompatible linear polysaccharide [30] and carboxymethylcellulose (CMC), an anionic water-soluble polysaccharide derived from cellulose were chosen as the two dominant polymer components of the coatings. The electrospun (ES) and 3D-printed coatings were loaded with clindamycin (CLIN), which is a bacteriostatic lincosamide known for its efficacy against streptococci, staphylococci, pneumococci, and Bacteroides species [31]. The coated and functionalised TiAlV and AISI 316LVM substrates were characterised by attenuated total reflectance–Fourier transform infrared spectroscopy (ATR-FTIR), field emission scanning–electron microscopy (FESEM), and atomic force microscopy (AFM). The concentration of dissolved CLIN in the receptor medium was determined using UV-VIS spectrophotometry. Contact angle measurements, swelling rate, and biodegradability of the coatings were also assessed. The biocompatibility of the 3D-printed coatings on AISI 316LVM and TiAlV substrates was estimated by conducting an MTT (3(4,5 dimethylthiazolyl-2)-2,5-diphenyltetrazolium bromide) test on osteoblast cell culture (hFOB-1.19, ATCC CRL11372). In the final step of our research, in vitro release testing of CLIN from the 3D-printed coatings was performed.

## 2. Experimental

### 2.1. Materials and Methods

Clindamycin hydrochloride monohydrate (C_18_H_33_ClN_2_O_5_∙HCl∙H_2_O) was purchased from Alfa Aesar, Haverhill, MA, USA. Sodium alginate (ALG; average mean molar mass *M*_w_ ≈ 80,000 g/mol), dimethyl sulfoxide (DMSO; purity ≥ 99.0%), 30 wt % nitric acid, calcium chloride (≥96.0%), carboxymethyl cellulose (CMC-3D, used for 3D printing; *M*_w_ ≈900,000 g/mol), and phosphate-buffered saline (PBS; pH 7.4 at 25 °C) were obtained from Sigma-Aldrich, St. Louis, MO, USA. The tetrazolium salt MTT (3(4,5 dimethylthiazolyl-2)-2,5-diphenyltetrazolium bromide) and carboxymethyl cellulose (CMC-ES, used for electrospinning, *M*_w_ ≈ 700,000 g/mol) were purchased from Sigma-Aldrich, Germany. Polyethylene oxide (PEO, *M*_w_ ≈ 600,000 g/mol) was supplied by ACROS, UK. Cellulose nanofibrils suspension (NFC, 3% (*w*/*v*)) was provided by The Process Development Center, University of Maine, Orono, ME, USA.

AISI 316LVM stainless steel and TiAlV were purchased from Tiger International, Shanghai, China, and Goodfellow Materials Ldt, Huntingdon, UK, respectively. Absolute ethanol was obtained from Honeywell (Charlotte, NC, USA). Ethanol (95 wt %) was purchased from Carlo Erba Reagents (Milan, Italy). Advanced Dulbecco’s Modified Eagle’s Medium (Advanced DMEM) and Fetal Bovine Serum (FBS) were purchased from Gibco (Grand Island, NY, USA). Human-derived skin osteoblasts hFOB 1.19 (ATCC CRL-11372) were purchased from the American Type Culture Collection (ATCC, Manassas, VA, USA). Ultra-pure water (18.2 MΩ cm at 25 °C) was produced with the Milli-Q ^®^ system (EMD Millipore Corporation, Burlington, MA, USA) and used for all the experiments.

### 2.2. Sample Preparation

#### 2.2.1. Preparation of Metal Substrates

Substrates in the shape of discs (diameter of 15 mm) made of AISI 316LVM (2 mm thickness) and TiAlV (1 mm thickness) were ground under a stream of water using 500-grit SiC papers (supplied by Struers, Ballerup, Denmark) until the surface was covered with a unidirectional scratch pattern. To minimise abrasion, the grinding direction was changed several times by turning the sample through 90°. In the next step, the substrates were cleaned in an ultrasonic bath containing 50% ethanol/50% ultra-pure water (V/V) solution. Then, AISI 316LVM substrates were passivated in 30 wt % nitric acid for 1 h. Both types of substrates (AISI 316LVM and TiAlV) were thoroughly rinsed with ultra-pure water, dried under a stream of air, and used to prepare ES and 3D-printed coatings.

#### 2.2.2. Preparation of the ES Coatings

The formulation for electrospinning was prepared by adding solutions of PEO (5% *w*/*v*) and CMC (7% *w*/*v*) to absolute ethanol in a volume ratio of 45:45:10 (PEO:CMC:EtOH). For the electrospinning of CLIN-functionalised nanofibers, CLIN was dissolved in the exact same solution mixture to yield a 1 mg/mL CLIN solution. Before electrospinning, the viscosity, conductivity, and surface tension of solutions were measured. Viscosity measurements were performed by using a Fungilab rotational viscometer (Smart series, Barcelona, Spain). Conductivity measurements were performed by using a SevenMulti™ S47 (Mettler Toledo International, Inc., Greifensee, Switzerland). Surface tension was determined by using a Tensiometer Krüss K-12 (KRÜSS GmbH, Hamburg, Germany). All measurements were performed at room temperature. The results are presented in Table 1.

Needle-free electrospinning was performed using a Nanospider™ NS500 (Elmarco, Liberec, Czech Republic) equipment. Parameters for the electrospinning process were as follows: the distance between collecting and feeding cylindrical electrodes was 160 mm, the working voltage was 60 kV, the speed of the rotating cylindrical electrode was set at 3.8 rpm, and the duration was 7 min. The room temperature during electrospinning was 21.5 ± 0.5 °C, and the relative humidity 33 ± 1%.

Photographs of the electrospun samples are shown in Figure S1b of the Supplementary document.

#### 2.2.3. Preparation of the 3D-Printed Coatings

Two different hydrogels were prepared for the 3D printed coatings. The first contained 6.0 wt % ALG, 6.0 wt % CMC, and 1.5 wt % NFC. The second hydrogel formulation differed only in the addition of CLIN and contained 6.0 wt % ALG, 6.0 wt % CMC, 1.5 wt % NFC, and 2.0 wt % CLIN. The coatings were 3D-printed onto AISI 316LVM and TiAlV substrates using a VitaPrint 3D bioprinter (IRNAS, Maribor, Slovenia). To print an optimal scaffold, various printing parameters (e.g., printing speed, extrusion pressure, printing temperature, the distance between the substrate and the extrusion nozzle, etc.) were tested. Extrusion nozzles (Nordson EFD, Westlake, OH, USA) with a 0.25 mm diameter were used for printing all samples. The scaffolds had 6 layers with a 16 mm diameter, a total height of 1 mm, and a 30% pore density, whereas respective succeeding layers were deposited in a 0°/90° fashion. All scaffolds were additionally cross-linked after printing using a 5 wt % CaCl_2_ solution. Finally, the samples were dried for 24 h at 37 °C in a controlled atmosphere containing 5 wt % CO_2_.

Photographs of the 3D-printed samples are shown in Figure S1a of the Supplementary document.

### 2.3. Coating Characterisation Techniques

The ATR-FTIR measurements were performed using an Agilent Cary 630 FTIR spectrometer (Santa Clara, CA, USA). The spectra were collected in the range of 4000–650 cm^–1^ at room temperature. The scans were performed on 3 different places in 3 repetitions.

For morphology studies of the ES and the 3D-printed coatings, a field emission scanning electron microscope (FE-SEM, Supra 35 VP, Carl Zeiss, Jena, Germany) at an acceleration voltage of 1 keV at room temperature was employed. All samples were attached on a double-sided adhesive carbon tape (SPI Supplies, West Chester, PA, USA). The freely available NIH image analysis software ImageJ 1.52r (NIH, Bethesda, MD, USA) [32] was used to calculate the electrospun fiber diameters. For this purpose, at least ten representative fibers were chosen from the image, and the results are presented as average diameters with standard deviations.

The topography and roughness parameters of the ES and the 3D-printed coatings were characterised by AFM in tapping mode with a Keysight 7500 AFM multimode scanning probe microscope (Keysight). The images were scanned using silicon cantilevers (ATEC-NC-20, Nanosensors, Wetzlar, Germany) with a resonance frequency of 210–490 kHz and a force constant of 12–110 Nm^−1^ [33]. All measurements were performed at room temperature. Images of (10 µm)^2^ and (1 nm)^2^ with a resolution of 512 × 512 pixels were recorded for all samples.

Static contact angles (CA) of ultra-pure water droplets were measured using an OCA15+ contact angle measurement system (Dataphysics, Filderstadt, Germany). The drop volume was 3 μL. All measurements were conducted in three repetitions at room temperature.

### 2.4. Swelling

The swelling kinetics of the 3D-printed coatings was investigated by the gravimetric method [21]. Freshly printed coatings were first dried for 24 h at 37 °C in a controlled atmosphere containing 5 wt % CO_2_. Then, the dried 3D-printed coatings were weighed and immersed in 25 mL of PBS (pH 7.4) at 37 °C. The coatings were removed from PBS at different immersion periods (0.5, 1, 2, 4, 6, 24, 48, and 120 h), carefully wiped dry with a filter paper, dried, weighed, and re-immersed in PBS. The swelling ratio at time *t* was calculated as the swelling ratio [%], according to Equation (1) [21]:

(1) $$\mathrm{swelling}\;\mathrm{ratio}\;\left[\%\right]=\;\frac{m_{t}\;-\;m_{0}}{m_{0}}\;\cdot \;100$$

where *m*_0_ is the initial dry mass and *m*_t_ is the dry mass of the swollen scaffold at time *t*. To determine *m*_0_ and *m*_t,_ the measurement was repeated three times.

### 2.5. Mass Loss

Mass loss experiments were performed according to Zhang et al. [34]. The dry 3D-printed coatings were weighed and then incubated in 25 mL of PBS (pH 7.4) at 37 °C. The 3D-printed coatings were removed from PBS at different immersion periods (1, 3, 7, and 10 days), washed with ultra-pure water, and wiped dry with a filter paper. The mass loss of the material was calculated according to Equation (2) [34]:

(2) $$\mathrm{mass}\;\mathrm{loss}\;\left[\%\right]=\;\frac{m_{t\left(2\right)}}{m_{0\left(2\right)}}\cdot \;100$$

where *m*_0(2)_ is the initial dry mass and *m*_t(2)_ is the dry mass of the coating at time *t*. To determine *m*_0(2)_ and *m*_t(2)_, the measurement was performed in three replications. Photographs of the experiment are shown in Figure S2b of the *Supplementary document*.

### 2.6. UV-VIS Spectrophotometric Determination of CLIN Concentration

The concentration of released CLIN in the receptor medium was determined using UV-VIS spectrophotometry. The UV-VIS molecular absorption spectra were recorded using a Cary 60 UV–VIS spectrophotometer (Agilent), and the quantification was performed at 210 nm (peak maximum). The concentrations were calculated according to the Beer–Lambert law. Due to sample withdrawal, sink conditions were ensured throughout the experiments. The accompanying dilutions during continuous sample withdrawal were accounted for in all calculations.

For determining the CLIN concentration, partial validation of the spectrophotometric method was performed by determining the limit of detection (LOD), limit of quantification (LOQ), linear concentration range, 90% and 95% confidence intervals, and sensitivity. Five calibration points were employed to determine the linear concentration range, and four replicate measurements were performed at each calibration point. Bartlett’s and Cochran’s test confirmed the homogeneity of the variances for the signals at calibration points for the reported linear concentration range. In contrast, the quantile-quantile plot confirmed the normality of the data distribution. The square of the correlation coefficient (*R*^2^) was 0.998, and the quality coefficient (*QC*) was 1.4%. The sensitivity (evaluated from the calibration curve slope) was 0.007 ± 0.001 ppm. The LOD and LOQ were determined using *s*_e_ (residual standard deviation) and *s*_b0_ (standard deviation of the intercept). The highest calculated value was taken as the method’s LOD/LOQ (a worst-case scenario of the method used). The determined LOD and LOQ were 2.33 and 7.77 ppm, respectively.

### 2.7. In Vitro Release Testing

In vitro release testing of CLIN from the 3D-printed coatings was performed using an Automated Transdermal Diffusion Cells Sampling System (Logan Instruments Corp., Logan System 912-6, Somerset, NJ, USA). The CLIN-functionalised coatings were slowly placed heads up into respective Franz diffusion cells. The receptor compartment was filled with PBS (0.01 M phosphate buffer, 0.0027 M potassium chloride, and 0.137 M sodium chloride, pH = 7.4 at 25 °C), while its temperature was maintained at 37 °C. During the dissolution testing, the medium was stirred continuously with a magnetic bar at 50 rpm and placed below the Franz diffusion cell [33]. Samples were collected for 3 days at the following time intervals: 5 min, 10 min, 20 min, 30 min, 1 h, 2 h, 3 h, 4 h, 5 h, 1 day, 2 days, 3 days. The concentration of the released CLIN in the receptor medium was quantified spectrophotometrically.

### 2.8. Cytotoxicity by MTT 3-(4,5-Dimethylthiazol-2-yl)-2,5-diphenyltetrazolium Bromide Reduction to Formazan

To test the biocompatibility of materials (ES and 3D-printed coatings on AISI 316LVM and TiAlV substrates), an MTT test on osteoblast cell culture (hFOB 1.19, ATCC CRL-11372) was performed. Cell viability testing was performed according to Mosmann [35]. First, the samples were sterilised under UV light for 30 min. In the second stage, the samples were incubated with osteoblast cell culture in P12 well plates for 24 h at 37 °C in an atmosphere containing 5 wt % CO_2_. The wells of the P12 well plates were filled with the cell culturing media (Advanced DMEM) and supplemented with 5 wt % FBS.

To perform the viability testing, each well of the P96 microtiter plates was filled with cell suspension containing 10,000 cells in media (advanced DMEM supplemented with 5 wt % FBS) and incubated overnight. The following day, samples and three different sample dilutions (1:2, 1:4, and 1:8) were moved to the P96 microtiter plates. After 24 h of incubation at 37 °C, the MTT assay was performed according to the manufacturer’s instructions. Photographs of the performed experiment are shown in Figure S2a of the Supplementary document.

## 3. Results and Discussion

### 3.1. Coating Characterisation

#### 3.1.1. ATR-FTIR Analysis

**Figure 1:**
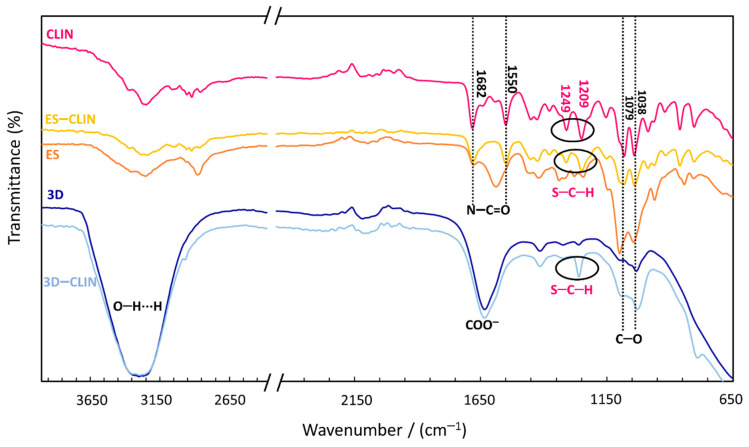
Attenuated total reflectance–Fourier transform infrared spectroscopy (ATR-FTIR) spectra of clindamycin (CLIN), the electrospun (ES), the ES-loaded with CLIN (ES–CLIN), the 3D-printed coating (3D), and the 3D-printed coating loaded with CLIN (3D–CLIN). CLIN-related peaks (S–C–H) in ES and 3D-printed polymer coatings are designated in ellipses.

ATR-FTIR spectroscopy was used to investigate the incorporation of the therapeutic agent (CLIN) into both types of polymer coatings. Therefore, the suitability of the as-prepared materials for further testing was determined (Figure 1). Several typical absorption signals characterise the ATR-FTIR spectrum of CLIN. Peaks corresponding to the N–C=O stretching appear at 1682 cm^−1^ and 1550 cm^−1^. The signals at 1038 cm^−1^ and 1079 cm^−1^ represent C–O stretching. The peaks at 1209 cm^−1^ and 1249 cm^−1^ were assigned to the S–C–H bending. An intensive and broad absorption band at 3200–3400 cm^−1^ for the 3D-printed coatings indicates the presence of O–H vibrations and the formation of hydrogen bonds, while the peak at around 1630 cm^−1^ most likely corresponds to the carboxylate in carboxymethyl cellulose [36]. The ATR-FTIR spectrum of CLIN-functionalised (ES and 3D-printed) polymer coatings showed no new functional peaks in either of the spectra, thus revealing that no new chemical bonds were formed between the drug and the polymer mixtures. The successful incorporation was confirmed by the presence of the peaks at 1209 cm^−1^ and 1249 cm^−1^ (assigned to S–C–H bending of the –SCH_3_ group in CLIN). The difference in the intensity of the CLIN-related peaks was most probably caused by the difference in the therapeutic agent’s concentration in the starting solutions.

#### 3.1.2. Morphology of the Coatings

**Figure 2:**
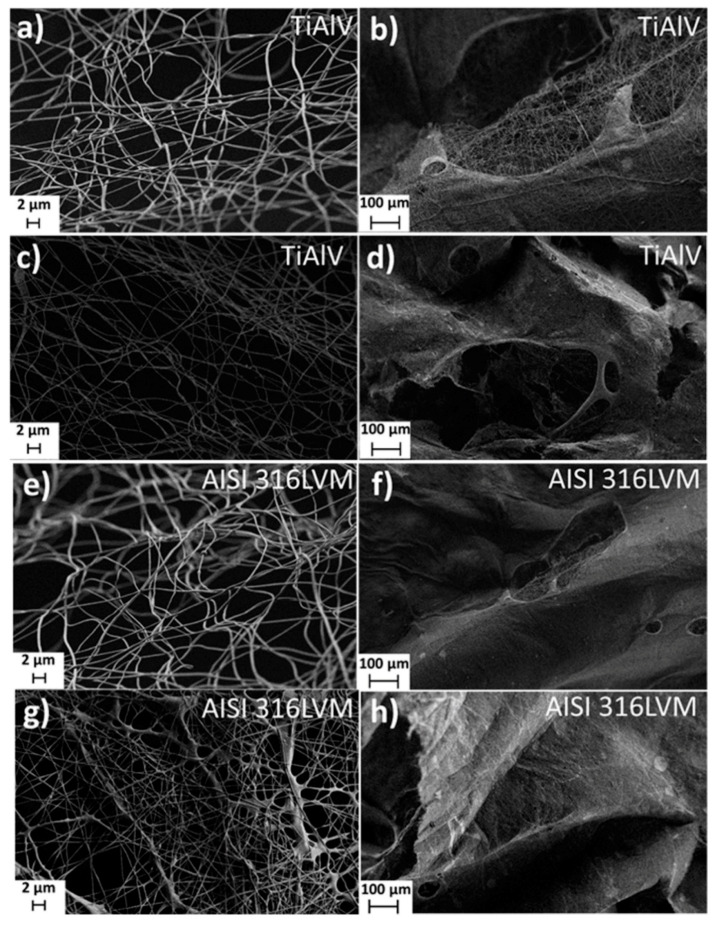
FE-SEM images of the non-functionalised (**a**,**b**,**e**,**f**) and CLIN-functionalised (**c**,**d**,**g**,**h**) ES on TiAlV (**a**–**d**) and ES on AISI 316LVM (**e**–**h**). Microphotographs were taken at different magnifications.

FE-SEM was used to study the overall morphology of the ES coatings (Figure 2). The analysis confirmed the presence of coherent systems of nanofiber structures (obtained by electrospinning). It further revealed that the type of metal substrate has no significant effect on the morphology of nanofibers. Loading of the ES coating with CLIN resulted in even, more homogeneous, and more densely distributed nanofibres. It is also evident that the addition of CLIN did not cause any structural defects (e.g., beads) that might occur due to local accumulation of the solvent [37]. Contrarily, according to the FE-SEM images obtained, the addition of CLIN had a positive effect on the electrospinning of nanofibers. This phenomenon might be explained by the slight difference in solvent properties for the electrospinning process, e.g., increased viscosity and conductivity of the CLIN-containing electrospinning solution (as seen in Table 1) [38]. The resulting fibers using various formulations for electrospinning were also compared regarding the average diameters. For this purpose, at least 10 fibers from each micrograph were analysed and included in the diameter calculation. As expected, CLIN’s addition into the electrospinning solution led to a slight increase in the average fiber diameter, which is evident in the case of coatings on both substrates (from 379 ± 97 nm to 521 ± 115 nm in the case of coatings on TiAlV, and from 408 ± 85 nm to 576 ± 234 nm in the case of coatings on AISI 316 LVM). Although the diameters seem to vary more between the samples based on the SEM micrographs, overall, the diameters of fibers produced from both formulations without CLIN (and from both with CLIN) are comparable.

**Figure 3:**
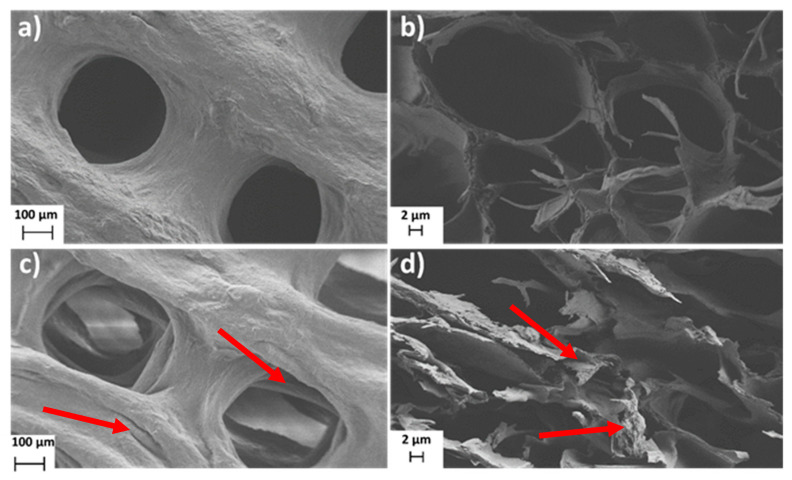
FE-SEM images of AISI 316LVM (**a**,**b**) and AISI 316LVM–CLIN (**c**,**d**). Microphotographs were taken at different magnifications and from different views ((**a**,**c**)—top view, (**b**,**d**)—side view). Red arrows show the additional morphological features (e.g., furrows) on the surface of the CLIN-loaded samples.

The FE-SEM technique was further used to study the morphology of the 3D-printed coating and CLIN’s influence on layer formation. Figure 3 shows FE-SEM images of the 3D-printed coatings on AISI 316LVM with (Figure 3c,d) and without (Figure 3a,b) CLIN. Since the substrate had no significant effect on the surface morphology of the 3D-printed coatings, the micrographs of the coated TiAlV substrates are not represented. The FE-SEM analysis of the 3D-printed coatings revealed the presence of nanofibrillated cellulose filaments on the surface of both types of coatings (AISI 316LVM and AISI 316LVM–CLIN), forming a rough surface favourable for cell growth. Additional furrows were noticed on the surface after the incorporation of CLIN (Figure 3c,d), which could stimulate cell growth even further. Moreover, a lack of CLIN clusters (e.g., crystals) on the 3D-printed surface suggests a homogeneous distribution of this therapeutic agent within the prepared coatings. The latter was also confirmed by AFM measurements (see below).

#### 3.1.3. AFM Measurements

**Figure 4:**
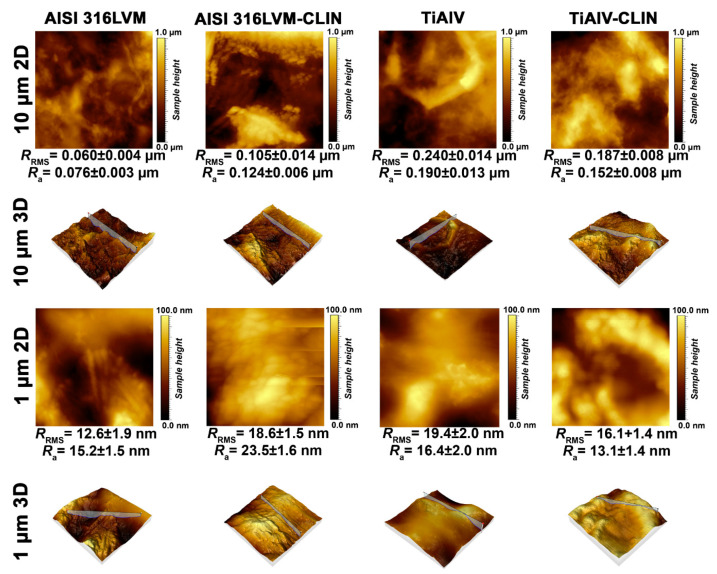
Surface morphology and roughness parameters of the 3D-printed coatings as measured by atomic force microscopy (AFM).

The surface of the 3D-printed samples was additionally analysed using the AFM technique. Figure 4 shows the topography of the 3D-printed coatings with and without CLIN on both types of metal substrates. The determined roughness parameters confirm that all samples exhibit a relatively smooth surface. Another important observation is that the functionalisation with CLIN has no significant effects on the morphology of the coatings, which is in agreement with the above findings obtained with the FE-SEM analysis that no CLIN clusters formed on the surface. According to the AFM measurements, the addition of CLIN results in an even distribution throughout the coating (or at least in the surface layer, which was assessed using AFM). The latter is crucial for the applicability of the studied systems, since localised clusters (bigger crystals of CLIN) could hinder the desired sustained drug release over time and/or lead to variations between repeated samples (i.e., inter-sample variability limits the applicability in biomedical applications). The values of roughness parameters further confirm the hypothesis on the homogeneous distribution of CLIN on the surface (and presumably in the bulk of the coating). These indicate a slightly increased roughness of the coating on AISI 316LVM after its functionalisation with CLIN. On the other hand, the values of roughness parameters for the coating on TiAlV alloy signify a slightly reduced roughness after functionalisation with CLIN. However, the sets of measurements for CLIN-functionalised coatings on both types of metal substrates show that the surface is smooth and homogeneous with roughness maxima in not more than tens of nm.

### 3.2. Contact Angle Measurements

Ideally, an orthopaedic implant should not only promote osteointegration [39] (the ability to bind to the surrounding bone, allowing incorporation of the graft at the host site [40]) but also encourage new bone deposition on its surface (osteoconduction) [29] to speed up the stabilisation process and to provide a seamless bone–implant interface [41]. It was shown that the osteointegration of Ti alloy and stainless-steel implants is related to hydrophilicity. Therefore, wettability (hydrophilicity) is an important property that affects the biocompatibility of materials in physiological environments [42]. The latter is usually determined using water contact angle measurements, which was also the method chosen for our study.

The results of contact angle measurements for all types of coatings are summarised in Table 2 and show that all samples’ angles are less than 90°, confirming that the surfaces are hydrophilic [43]. The measured contact angles show that the ES coatings have lower contact angles and are thus more hydrophilic than the 3D-printed coatings. The reason for this lies in the different composition of the polymeric solutions (among others, the inclusion of PEO). Moreover, the contact angles of the CLIN-functionalised coatings are lower than the contact angles of their non-functionalised equivalents, which might be explained by the hydrophilicity of this therapeutic agent.

### 3.3. Swelling and Mass Loss

**Figure 5:**
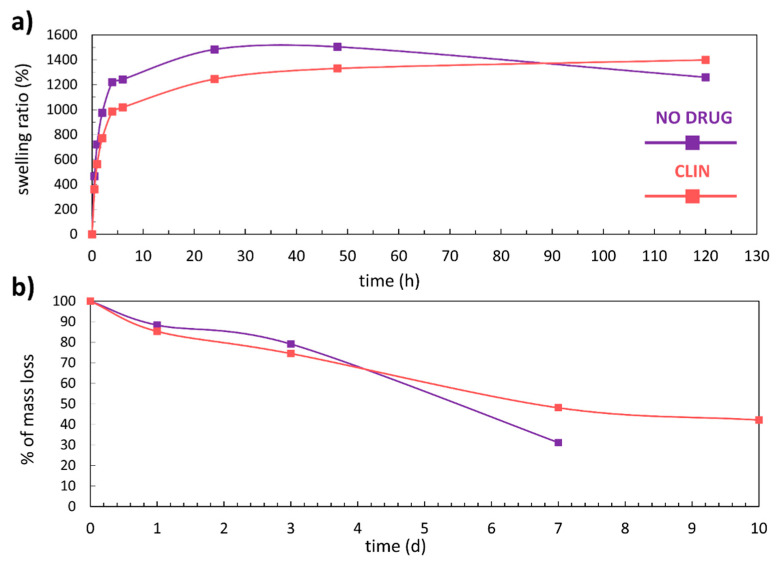
(**a**) Swelling kinetics of the 3D-printed coatings in phosphate-buffered saline (PBS) (pH 7.4) at 37 °C and (**b**) degradation curves (% of mass loss as a function of time) of the 3D-printed coatings in PBS (pH 7.4) at 37 °C.

The degree of swelling is influenced by the extent of cross-linking and the polymer’s chemical nature. It dictates many of the important properties in the design of hydrogels as drug delivery systems or for any other use [28]. Figure 5a and Table 3 show the results obtained in the experiments to determine the swelling rate of the 3D-printed coatings over 120 h. Both types of the 3D-printed coatings (non-functionalised and CLIN-functionalised) show a significant degree of swelling. The coating without the incorporated drug swelled for almost 1500% and reached equilibrium after 24 h. A slight loss of mass can be observed after 48 h. The further drop in the swelling rate at 120 h can be explained by the fact that the degradation process overpowered the fluid uptake. Alternatively, the decline in the swelling rate can be partly attributed to the repeated handling of the samples (to perform the measurements). The graph of the CLIN functionalised 3D-printed coating shows that the drug caused a noticeable decrease in the swelling rate in the first 48 h. From that point, an increase in the swelling rate can be observed, thus confirming that the functionalisation with CLIN improved the long-term moisture absorption properties of the 3D-printed coatings.

Biodegradability is an important characteristic of materials considered for various biomedical applications (e.g., drug delivery applications), even more so in the case of implantable materials [44]. For dissolution to occur, such material (in our case, a hydrogel) must absorb the surrounding aqueous solvent and interact with water via charge interactions or hydrogen bonding mechanisms [45]. Figure 5b shows the degradation curves for the non-loaded and the CLIN-loaded 3D-printed coatings, while the corresponding data are given in Table 4. The shapes of the curves signify an apparent mass loss upon the exposure to PBS at 37 °C. The functionalisation of the 3D-printed coating influenced the long-term stability of the coating and its degradation kinetics. When comparing the degradation level after 7 days for both types of coatings, one can notice that the mass loss of the non-loaded coating is 20% greater than the mass loss of the CLIN-loaded coating. Moreover, the non-loaded coating completely disintegrated after 7 days. On the other hand, only 60% of the CLIN-functionalised 3D-printed coating degraded after 10 days, which is encouraging for the potential clinical applicability of the system in the future.

The swelling and the degradation tests for the ES coatings could not be performed due to their decomposition almost immediately after immersion into PBS. Similar results were obtained for another of our ES materials, where only additional cross-linking steps led to a material stable enough for further testing [46]. Despite the other encouraging results for the ES samples, it was predicted that these coatings would not be able to compete with the performance of the 3D-printed coatings. This is also the reason why all the following results are presented for the 3D-printed coatings only.

### 3.4. MTT Test

Biocompatibility tests on relevant cell cultures represent an essential part of testing materials intended for use as a part of biomedical implants. Since our final goal is to incorporate the newly developed materials into hip implants, all tests were performed on cells with which implants come into contact during such applications (human osteoblasts). The purpose was to determine whether the materials impede osteoblastic cell growth and whether toxic products are formed during exposure (either through degradation or through local overdoses of the incorporated CLIN).

**Figure 6:**
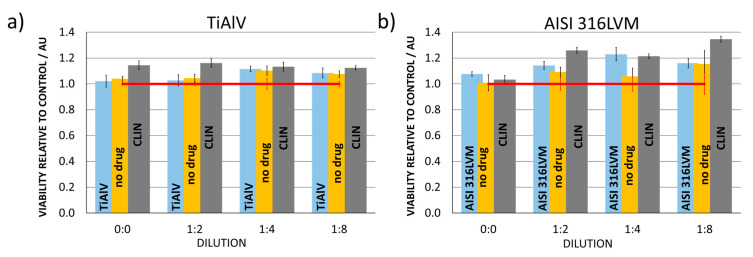
Osteoblast cell viability determination for (**a**) TiAlV substrate, 3D-printed coating on TiAlV substrate (no drug), and CLIN-loaded 3D-printed coating on TiAlV substrate (CLIN) and (**b**) AISI 316LVM substrate, 3D-printed coating on AISI 316LVM substrate (no drug), and CLIN-loaded 3D-printed coating on AISI 316LVM substrate (CLIN) using the MTT (3(4,5 dimethylthiazolyl-2)-2,5-diphenyltetrazolium) assay and the control (cell growth in the advanced DMEM and FBS medium indicated by red line). Error bars represent standard deviation.

Quantitative assessment of cell viability was performed using the MTT assay. The column charts in Figure 6 show that (regardless of the dilution) none of the materials used for the preparation of the functionalised 3D-printed coatings in this study decreases cell viability relative to control (cell growth in the advanced DMEM and FBS medium). Moreover, the relative cell viability was higher than for the control in all cases, confirming that the as-prepared 3D printed coatings are suitable for the intended application. Loading of the 3D-printed coatings with CLIN even enhanced cell viability on both substrates (TiAlV and AISI 316LVM), which confirms the suitability of the selected therapeutic agent.

### 3.5. In Vitro Release Study

**Figure 7:**
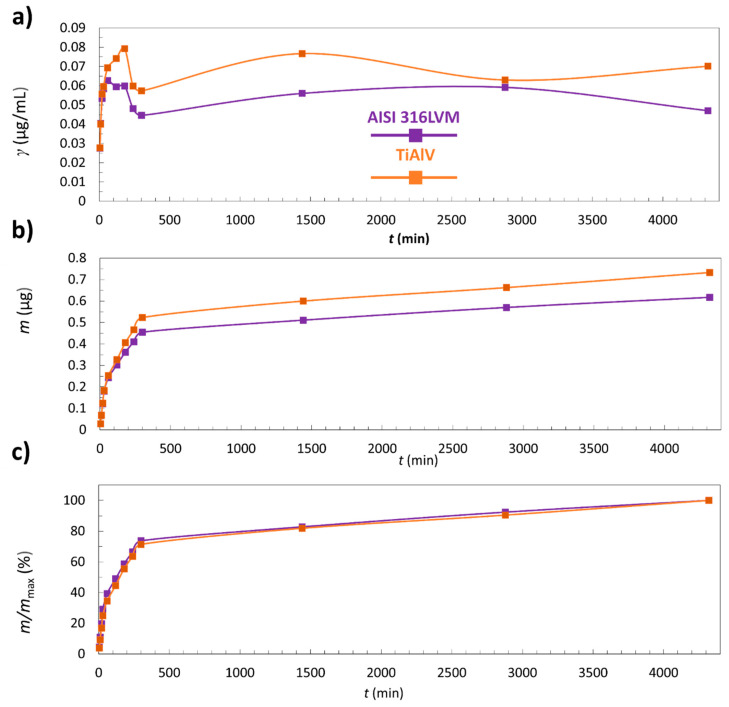
Results from the in vitro CLIN release testing of the 3D-printed coatings on AISI 316LVM and TiAlV: (**a**) the concentration of the released CLIN as a function of time (**b**) the cumulative mass of the released CLIN as a function of time, and (**c**) the mass fraction of the released CLIN as a function of time.

The results of in vitro release studies of CLIN from the 3D-printed coatings on both types of substrates (AISI 316LVM and TiAlV) are summarised in Figure 7.

Figure 7a represents the concentration of CLIN release from the 3D-printed coatings as a function of time. After the initial rapid release, a decrease in concentration for coatings on AISI 316LVM and TiAlV occurs. This can be explained by the fact that most of the incorporated CLIN was released in the first 60 min. It must be pointed out that the decrease in concentration occurs partially due to sample withdrawal, followed by the addition of fresh medium, resulting in dilution of the release medium (as explained in Section 2.6 and Section 2.7).

Figure 7b shows cumulative released CLIN masses from the coated AISI 316LVM and TiAlV substrates as a function of time. The results show that the 3D-printed coatings on both substrates follow the same release mechanism where the initial burst of CLIN from the coating in the first 60 min is followed by a swift release until the 300th minute. After that, the release from the coating becomes slow—a plateau is reached.

Figure 7c shows that after 24 h, only 82% of CLIN was released from the coating on TiAlV and 83% from the coating on AISI 316LVM.

The drug-release kinetics is an essential governing factor in infection management, which is true regardless of the type of administration (local or systemic). Ideally, a drug-release profile should consist of two phases—a bolus drug release of high concentrations of antibiotics to immediately reach the antibacterial effect, followed by a sustained drug release (above the minimum inhibitory concentration) that continuously “neutralises” newly grown bacteria. With such a bimodal activity and local drug release, biofilm cannot even form [19]. Therefore, the release of CLIN from the 3D-printed coating in a burst-like manner is an encouraging result. Furthermore, the fact that only approximately 80% of the drug got released from the 3D-printed coating after 24 h confirms the potential of the newly developed coatings for applications in hip prostheses. The 24 h time frame corresponds to the current guidelines of post-operative antibiotic prophylaxis in total hip and knee arthroplasty [47], and total joint arthroplasty [48]. After the initial local antibacterial effect, systemic therapy can be applied to lower the potential of infections even further.

## 4. Conclusions

In this study, novel electrospun (ES) and 3D-printed coatings were prepared on two different metal substrates (AISI 316LVM stainless steel and Ti90Al6V4 alloy). The ES coatings consisted of carboxymethyl cellulose (CMC) and polyethylene oxide (PEO). In contrast, the 3D-printed coatings in the form of hydrogels were prepared from a polymeric mixture containing CMC, nanofibrillated cellulose, and alginate (ALG). Both types of coatings were further loaded with the antibiotic clindamycin (CLIN).

ATR-FTIR surface analysis confirmed the successful incorporation of CLIN into the coatings prepared by both methods. FE-SEM was used to study the morphology of the CLIN-functionalised and non-functionalised ES and the 3D-printed coatings on AISI 316LVM and Ti90Al6V4 substrates. It was found that ES coatings retained the uniform and homogeneous distribution of nanofibers even after functionalisation with the therapeutic agent. Moreover, AFM measurements confirmed the homogeneous distribution of CLIN on the 3D-printed coatings. Contact angle measurements determined the hydrophilic character of the 3D-printed and ES materials. Due to almost immediate decomposition of ES scaffolds in the PBS medium, the study of ES coatings was limited. Therefore, swelling tests, degradation tests, in vitro release, and viability testing could only be performed for the 3D-printed scaffolds. The results showed that the CLIN-functionalised coatings have better moisture absorption properties than the non-functionalised coatings. Mass loss experiments determined the biodegradability of the 3D-printed coatings. Only 60% of the CLIN-functionalised 3D-printed coating degraded after 10 days. On the other hand, the non-functionalised coating was found to completely disintegrate after 7 days. Our findings of in vitro release study show that the CLIN-functionalised 3D-printed coatings provide continuous release of antibiotic from the matrix, which follows an initial burst release. This is suitable for an immediate antibacterial effect. The complete release of the antibiotic was noticed after 3 days, which is desirable for potential applications in hip prosthesis coatings. None of the prepared coatings (including the substrate itself) impede osteoblastic cell growth and they do not release toxic products, as successfully demonstrated by the MTT assay.

Based on the results obtained in this study, novel 3D-printed CLIN-functionalised coatings show great potential for applications in orthopaedic implants that require long-term release of antibiotics to successfully prevent post-operative infections. On the other hand, the proposed ES coating would need a further cross-linking step to improve their long-term stability and provide a viable option as implant coatings.

Our future research will focus on studying the suitability of the presented coatings for the functionalisation with other antibacterial agents. Moreover, optimisation will be performed with the aim of developing systems that enable different time release profiles of the incorporated therapeutic components.

## Figures and Tables

**Table 1:** Viscosity, conductivity, and surface tension of solutions for electrospinning.

	No Drug Solution	CLIN Containing Solution
Conductivity (µS/cm)	4157.00	4677.00
Viscosity (mPas)	874.90	878.90
Surface tension (mN/m)	53.22	38.46

**Table 2:** Contact angles for the ES and the 3D-printed coatings with and without CLIN on AISI 316LVM and TiAlV. The results are given as average values ± standard deviation for the replicate measurements.

Metal Substrate	AISI 316LVM [°]		TiAlV [°]	
	no drug	CLIN	no drug	CLIN
3D-printed coating	56.3 ± 2.4	53.9 ± 5.7	68.1 ± 0.3	60.1 ± 2.3
ES coating	47.5 ± 2.3	44.8 ± 3.2	54.8 ± 3.2	49.6 ± 3.1

**Table 3:** The results obtained in the experiments to determine the degree of swelling. The results are given as average values ± standard deviation for the replicate measurements.

	Non-Functionalised		CLIN-Functionalised	
Time (h)	Weight (g)	Swelling Ratio (%)	Weight (g)	Swelling Ratio (%)
0	0.09551 ± 0.00046	0.0	0.12603 ± 0.00310	0.0
0.5	0.54001 ± 0.00679	465.4 ± 6.8	0.58152 ± 0.06129	361.4 ± 38.1
1	0.78395 ± 0.01856	720.8 ± 16.4	0.83434 ± 0.06214	562.0 ± 32.6
2	1.02736 ± 0.00551	975.7 ± 7.5	1.09756 ± 0.06776	770.8 ± 33.2
4	1.26217 ± 0.03977	1221.6 ± 44.6	1.36763 ± 0.06322	985.1 ± 4.4
6	1.28304 ± 0.06441	1243.3 ± 64.2	1.41080 ± 0.05592	1019.4 ± 23.5
24	1.51262 ± 0.06430	1483.8 ± 68.7	1.69674 ± 0.09510	1246.3 ± 43.9
48	1.53228 ± 0.06408	1504.4 ± 67.0	1.80347 ± 0.08884	1330.9 ± 49.1
120	1.29933 ± 0.04868	1260.4 ± 46.8	1.88958 ± 0.02472	1399.3 ± 35.3

**Table 4:** The data for the plotting of the degradation curves in Figure 5b. The results are given as average values ± standard deviation for the replicate measurements.

	Non-Functionalised		CLIN-Functionalised	
Time (days)	Weight (g)	Degradation (%)	Weight (g)	Degradation (%)
0	0.09050 ± 0.00131	100.0	0.12821 ± 0.00165	100.0
1	0.07997 ± 0.00557	88.4 ± 6.2	0.10938 ± 0.00145	85.3 ± 1.1
3	0.07162 ± 0.00806	79.1 ± 8.9	0.09553 ± 0.00445	74.5 ± 3.5
7	0.02825 ± 0.00057	31.2 ± 0.6	0.06171 ± 0.00227	48.1 ± 1.8
10	/	/	0.05409 ± 0.00017	42.2 ± 0.1

## Data Availability

The data presented in this study are available on request from the corresponding author.

## Supplementary Materials

The following are available online at https://www.mdpi.com/1996-1944/14/6/1464/s1, Figure S1: Photographs of: (a) 3D printed samples, and (b) electrospun samples. NO differences between the samples are directly observable. Figure S2: Photographs of experiments: (a) cell testing, and (b) degradation studies.
