# Supplementary material for: Clindamycin-Based 3D-Printed and Electrospun Coatings for Treatment of Implant-Related Infections
Source: Materials (Basel). 2021 Mar 17;14(6):1464. doi: 10.3390/ma14061464 (PMC8002500; doi:10.3390/ma14061464)
Supplement: Supplementary file 1 [file materials-14-01464-s001.pdf]

SUPPLEMENTARY DOCUMENT

Clindamycin based 3D Printed and Electrospun Coatings for Treatment of Implant-Related Infections

Tina Maver[1,2,*], Tinkara Mastnak[3], Mihela Mihelič[3], Uroš Maver[1,2] and Matjaž Finšgar[3,*]

[1] Institute of Biomedical Sciences, Faculty of Medicine, University of Maribor, Taborska ulica 8, SI-2000 Maribor, Slovenia
[2] Department of Pharmacology, Faculty of Medicine, University of Maribor, Taborska ulica 8, SI-2000 Maribor, Slovenia
[3] Faculty of Chemistry and Chemical Engineering, University of Maribor, Smetanova ulica 17, 2000 Maribor, Slovenia

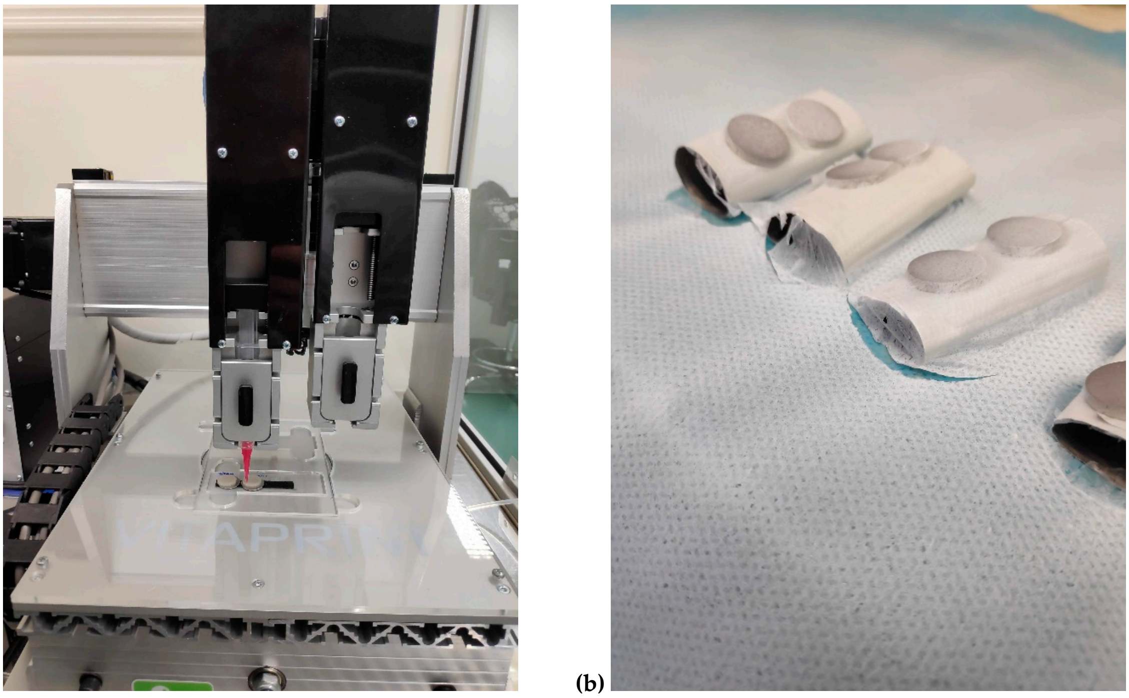

(a) (b)

Figure S1. Photographs of: (**a**) 3D printed samples, and (**b**) electrospun samples. NO differences between the samples are directly observable.

SUPPLEMENTARY DOCUMENT

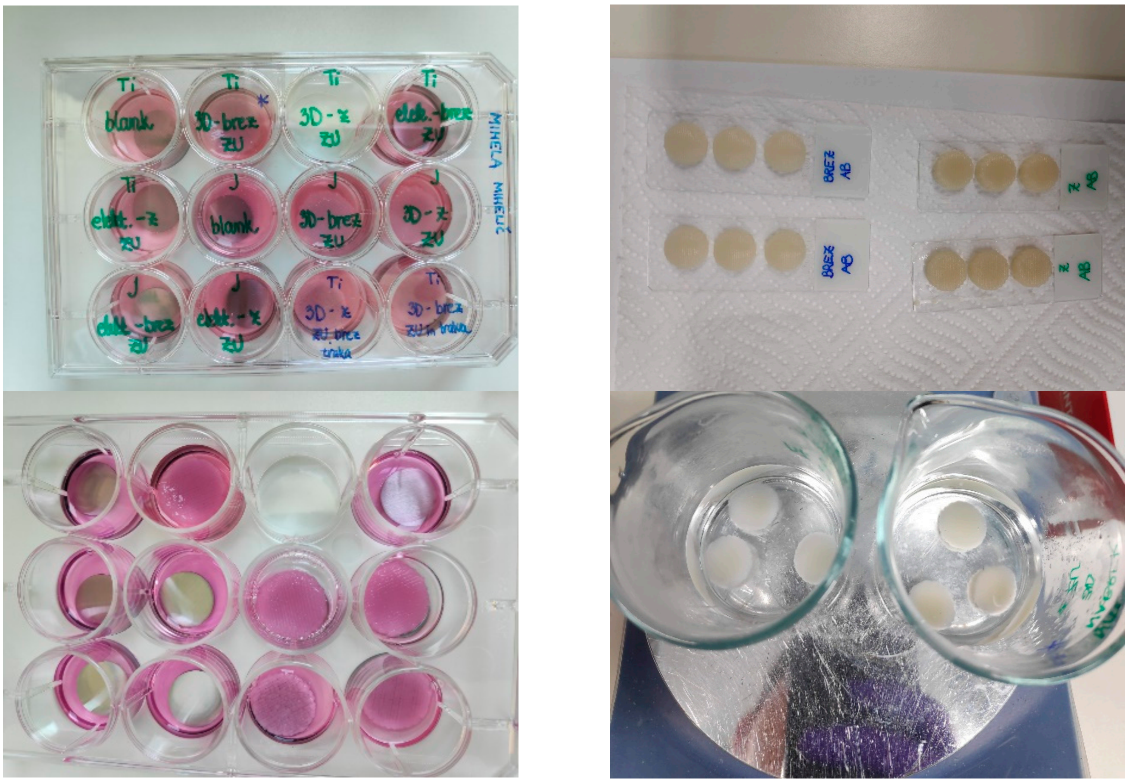

(a) (b)

Figure S2. Photographs of experiments: (**a**) cell testing, and (**b**) degradation studies.
